# Performance evaluation of the smartphone-based AI cough monitoring app - Hyfe Cough Tracker against solicited respiratory sounds

**DOI:** 10.12688/f1000research.122597.1

**Published:** 2022-07-01

**Authors:** Mindaugas Galvosas, Juan C. Gabaldón-Figueira, Eric M. Keen, Virginia Orrillo, Isabel Blavia, Juliane Chaccour, Peter M. Small, Gerard Giménez, Simon Grandjean Lapierre, Carlos Chaccour

**Affiliations:** 1 Research and Development Department, Hyfe Inc., Wilmington, Delaware, USA; 2 Department of Microbiology and Infectious Diseases, Clinica Universidad de Navarra, Pamplona, Spain; 3 School of Pharmacy and Nutrition, University of Navarra, Pamplona, Spain; 4 Department of Global Health, University of Washington, Seattle, Washington, USA; 5 Immunopathology Axis, Research Center, University of Montreal Hospital Center, Montreal, Canada; 6 Department of Microbiology, Infectious Diseases and Immunology, University of Montreal, Montreal, Canada; 7 Centro de Investigación Biomédica en Red de Enfermedades Infecciosas, Madrid, Spain; 8 ISGlobal, Hospital Clinic, University of Barcelona, Barcelona, Spain

**Keywords:** cough, artificial intelligence, cough monitoring, cough counting, hyfe, hyfe cough tracker

## Abstract

**Background** : Emerging technologies to remotely monitor patients’ cough show promise for various clinical applications. Currently available cough detection systems all represent a trade-off between convenience and performance. The accuracy of such technologies is highly contingent on the clinical settings in which they are intended to be used. Moreover, establishing gold standards to measure this accuracy is challenging.

**Objectives**: We present the first performance evaluation study of the Hyfe Cough Tracker app, a passive cough monitoring smartphone application. We evaluate performance for cough detection using continuous audio recordings and cough counting by trained individuals as the gold standard. We propose standard procedures to use multi-observer cough sound annotation from continuous audio recordings as the gold standard for evaluating automated cough detection devices.

**Methods** : This study was embedded in a larger digital acoustic surveillance study (clinicaltrial.gov NCT04762693). Forty-nine participants were included and instructed to produce a diverse series of solicited sounds in 10-minute sessions. Simultaneously, continuous audio recording was performed using a MP3 recorder and two smartphones running Hyfe Cough Tracker app monitored and identified cough events. All continuous audio recordings were independently labeled by three medically-trained researchers.

**Results** : Hyfe Cough Tracker app showed sensitivity of 91% and specificity of 98% with a very high correlation between the cough rate measured by Hyfe and that of human annotators (Pearson correlation of 0.968). A standardized approach to establish an acoustic gold standard for identifying cough sounds with multiple observers is presented.

**Conclusion:** This is the first performance evaluation of a new smartphone-based cough monitoring system. Hyfe Cough Tracker can detect, record and count coughs from solicited cough-like explosive sounds in controlled acoustic environments with very high accuracy. Additional steps are required to validate the system in clinical and community settings.

## Introduction

Cough is consistently ranked as one of the most common reasons for seeking medical attention. ^1^^,^^2^ Acute cough frequently indicates new-onset and potentially contagious respiratory infection, ^3^ while chronic cough can be an important cause of discomfort and disability affecting quality of life. ^4^^,^^5^ In current medical practice, objective cough assessment can only occur during face to face interaction with the patient in the context of in- or outpatient visits, effectively making the symptom invisible to the health care provider outside the medical settings. To assess cough in ambulatory settings, health care providers rely on questionnaires and patient-reported outcomes, which are subject to patients’ self-perception, cough tolerance and recall bias. ^6^^,^^7^ While different systems for automated cough detection have been developed in the last decade, ^8^^,^^9^ they depend on wearable microphones, or spirometers, ^10^ and their adoption is limited by cost, portability and privacy concerns given the need for continuous sound recording. Recent advances in artificial intelligence (AI) allow the monitoring of cough in a non-obtrusive way using smartphones or other wearable digital devices. ^6^^,^^11^^–^^14^ Unobtrusive and privacy preserving passive cough monitors could revolutionize clinical practice and research in the field of respiratory diseases.

Longitudinal monitoring of cough is particularly attractive for the evaluation of disease progression, or treatment response, as well as in clinical trials where trends in cough rates is an outcome of interest. Longitudinal cough monitoring also opens the door to population-wide capture of cough-signals as a surrogate marker of respiratory diseases epidemiology. ^14^

Evaluating cough and its patterns with limited recording periods (e.g., 24 h) can be misleading, in particular, if only small changes in cough frequency are captured over the limited 24 h recording and in cases that have high variance of cough counts. ^12^ However, the nature and volume of data generated with protracted monitoring raises new challenges in technology validation. A central challenge in this work is establishing a gold standard against which automated devices’ performance can be evaluated.

In this study, we present the accuracy of Hyfe Cough Tracker app (henceforth referred to as Hyfe), a smartphone-based automated cough monitor that uses a convolutional neural network (CNN) to differentiate coughs from other explosive sounds. ^13^ In this “in-vitro” performance evaluation, we use solicited sounds in a controlled acoustic environment as the first step towards clinical validation. We also propose a standard operating procedure (SOP) to appropriately label cough sounds from continuous audio recording.

## Methods

### Automated cough detection system

Hyfe is a software application for patient use, freely available for use on Android and iOS smartphones. It continuously monitors ambient sound and employs a two-step process to (1) detect explosive cough-like sounds, record a 0.5 second sound snippet which is sent to a cloud server where (2) a CNN assigns a cough prediction score (0 to 1) to each sound. For this study, a minimal score threshold of 0.85 was used for classifying a peak sound as a cough. Within this study, Hyfe (Version acl 1.24.4) was installed on smartphones (Motorola G30, Motorola, Inc, Chicago, IL, USA) running Android operating system version 11 (Google, LLC, Mountainview, CA, USA).

To assess the accuracy of Hyfe, continuous recording using a MP3 recorder (Sony ICD-PX470, Sony, Tokyo, Japan) and manual labeling of cough by medically trained listeners was used as the gold standard.

### Study design

This performance evaluation study was conducted at the University of Navarra, Spain, between September to November 2021 and was nested in a larger cohort study ( Clinicaltrials.org NCT04762693). ^12^ Both the main and the nested study received approval by the Medical Research Ethics Committee of the chartered community of Navarra (PI_2020/107). Students and staff from the university of Navarra were invited to participate via email. All participants were aged 18 or older and signed informed consent. Baseline respiratory symptoms were not considered for inclusion. Participants were asked to produce a series of solicited sounds by reading a provided script, while being recorded with an MP3 recorder and monitored by Hyfe on two identical smartphones. The phones and recorder were placed on a table at approximately 50 cm from the participants, with microphones oriented towards them.

A pre-generated computer script instructed participants to produce a series of 46 sounds, of which 18 were coughs, the rest consisted of solicited sneezes, throat clearings, spoken letters or words in the same 10 minutes. Some sounds were requested while reading out loud a literary text (in Spanish). Outside the reading, solicited sounds were separated from one another by at least five seconds of ambient silence. There were five different versions of the script, each one presenting a different sequence of instructions, and the version shown to each participant was randomly selected using a computer-generated sequence at the beginning of each session. Recording sessions occurred in a quiet room and lasted approximately 10 minutes. The time at which individual sounds were produced was automatically recorded in every session.

Three medically trained researchers listened to individual recording sessions using Audacity (Audacity team (2021). Audacity(R): Free Audio Editor and Recorder [Computer application]. Version 3.1.3). ^15^ Coughs were manually annotated using digital audio recordings and visual audio wave representation. It was previously shown that ambulatory cough counts from audio recordings have great agreement with patient video recordings, and that digital audio recordings could hence be considered as the gold standard in validating novel cough monitoring tools. ^16^^,^^17^ Each sound was labeled using a 4-tier system defined in the SOP, which was developed for cough annotation in continuous audio recordings. In brief, sounds were classified as 0 = definitely not a cough, 1 = disputable cough (i.e., someone could consider the sound as a cough), 2 = definite cough but distant/muffled/obstructed, 3 = definite cough. Labels were made using Audacity and exported as text files for analysis. Labellers were blinded to the classification made by Hyfe and other listeners but knew a participant’s age and gender. Sounds labeled unanimously as a number 3 (“definite cough”) by all the human listeners were considered true coughs.

### Sample size

We estimated that at least 385 sounds would be required to observe a 90% sensitivity and 85% specificity, with a cough prevalence of 40% (39% of solicited sounds in the script were coughs), a precision of 5%, and a dropout rate of 10%. ^18^^,^^19^

### Data processing and analysis

Labels created by listeners (in Audacity) and Hyfe detected coughs were manually synchronized to within two seconds of each other. Synchronization was carried out for each phone and each session separately by identifying the time offset that would align Hyfe detections with the labels and adjusting the Hyfe detection timestamps accordingly. Offsets were estimated first using a subroutine in R that iteratively tests the offset-error produced by a wide variety of values, then manually reviewing and adjusting those automatic offsets as needed.

For the performance analysis, each recording session was divided into seconds. Seconds in which at least one explosive cough-like sound was labeled by a human listener (categories 1, 2, or 3) were pooled and defined as “cough-like-seconds”. Individual labels, which were annotated by the listeners, occurring within one second of each other were treated as a single label, and included as a single “cough-second”. Similarly, seconds in which only non-cough sounds occurred (category 0), were identified as “non-cough seconds”.

Hyfe detections on each phone were also pooled into cough-seconds using a similar method: all detected explosive sounds occurring within a one-second period were treated as a single detection; if multiple explosive sounds occurred within a cough-second, the highest cough prediction score among all explosive phases was used as the prediction score for the cough-second.

**Figure 1.**
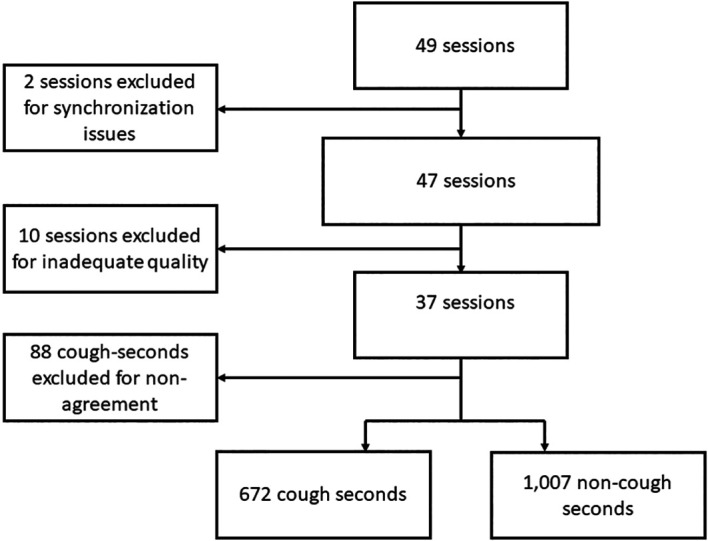
Study flow chart.

All recording seconds were considered as distinct analysis units. Seconds for which there was disagreement between the three human listeners were excluded from the final analysis. Similarly, 10-minute sessions in which less than 10 cough-seconds were unanimously labeled as such by human listeners were considered of inadequate quality and excluded. ( Figure 1) True positives (TP) assessments were defined as those cough-seconds detected by Hyfe, and unanimously classified as category 3 by all human listeners. False positives (FP) were defined as seconds in which coughs did not actually occur, but were incorrectly detected by Hyfe. A pooled sensitivity and specificity value for each phone was obtained by aggregating the cough- and non-cough seconds labeled and detected by each phone throughout all sessions included in the analysis. The fraction of TP among cough-seconds was calculated (sensitivity), as well as the fraction of FP among all non-cough seconds, which was used to calculate the specificity, using the following formula: 1 - (FP/non-coughs) = Specificity.

Given inter-participant variation in ability to generate coughs and other sounds, the performance characteristics of Hyfe for each combination of phone and session were individually assessed and then used to calculate an average sensitivity and specificity in an exploratory sub-analysis.

All data processing and analysis was performed in R version 4.02 (R Core Team 2020) and the code used is available from GitHub and is archived with Zenodo. ^23^

This analysis further informed the SOP used by Hyfe to annotate coughs and cough-like sounds (sneezes and throat clears), leading to the most recent version - the 6-tier SOP for cough labeling in continuous audio recordings, which now also instructs to label the complete duration of target sounds.

Because the utility of a cough monitor is not in noting individual coughs but rather in tracking cough rates, we further analyzed these results to look at the overall performance of Hyfe to the human annotated gold standard. We cut the entire observation period for all participants into one-minute segments, then compared the gold standard (the number of coughs during that minute per the human annotator) against the tool (the number of cough detections per Hyfe).

## Results

In total, 49 recording sessions with individual participants of approximately 10 minutes each were carried out. Two sessions did not have enough labels or detections to allow adequate timestamp synchronization and were excluded. Ten sessions did not have at least 10 sounds unanimously labeled as coughs and were also excluded, leaving 37 sessions, with 672 unanimously-labeled cough-seconds, and 1,007 non-cough seconds for the final performance evaluation ( Figure 1).

**Figure 2.**
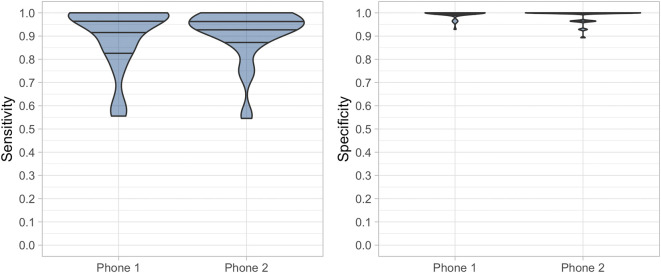
Performance of both phones through the 37 studied sessions. Sensitivity and specificity of Hyfe Cough Tracker assessed using solicited coughs.

The performance of Hyfe using both phones was similar in the pooled analysis, and is presented in Figure 2. Phone 1 yielded a sensitivity of 91.5% (95% CI: 89.2%-93.5%) and a specificity of 99.3% (95%CI: 98.6%-99.7%, Table 1), while phone 2 yielded a sensitivity of 92.55% (95% CI: 90.3%-94.4%) and a specificity of 98.7% (95% CI: 97.8%-99.3%, Table 1). The performance of both phones in individual sessions was also evaluated - the average sensitivity of the system in both phones and through the 37 sessions was 90.8% (SD = 11.6%). Specificity was high in both phones (range 93%-100% for phone 1, and 89%-100% for phone 2), with the mean specificity being 99.1% (SD = 1.9%).

**Table 1.** Comparative performance of both phones used.

		Human labels					
		Phone 1			Phone 2		
		Cough seconds	Non-cough seconds	Total	Cough seconds	Non-cough seconds	Total
Hyfe’s classification	Cough seconds	615	7	622	622	13	635
	Non-cough seconds	57	1000	1057	50	994	1044
	Total	672	1007	1679	672	1007	1679

**Figure 3.**
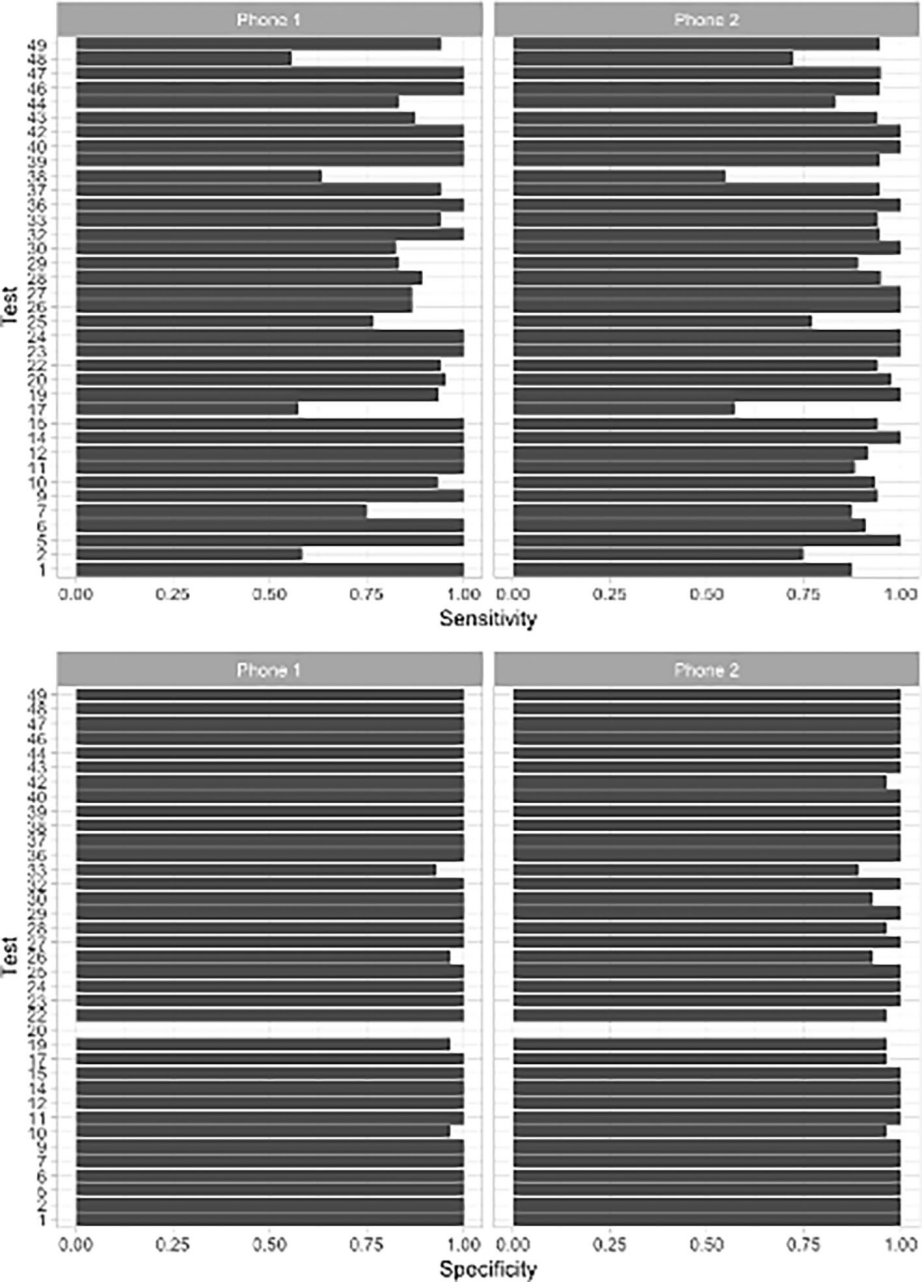
Performance of the Hyfe Cough Tracker in individual recording sessions.

**Figure 4.**
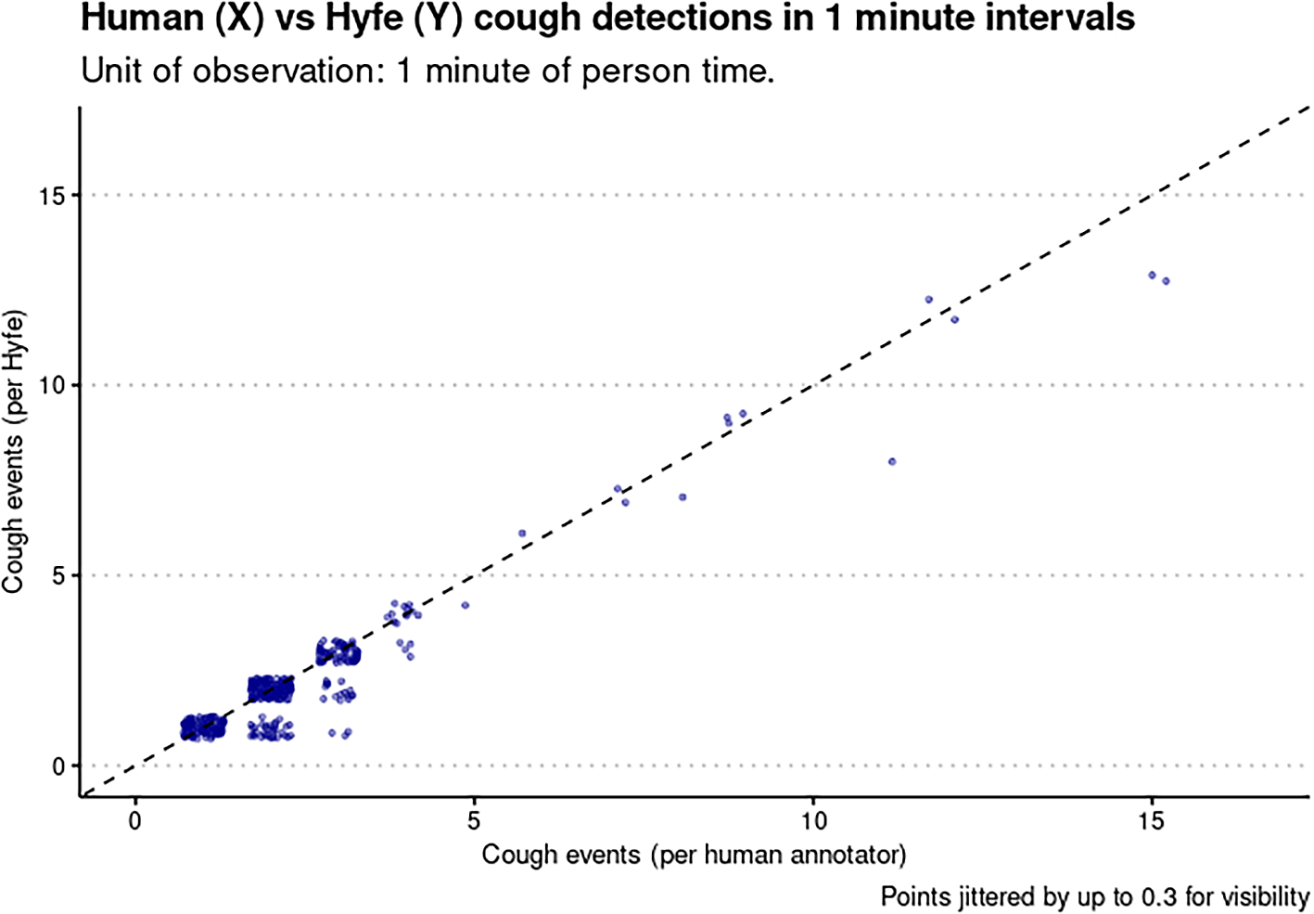
Correlation between the gold standard (human annotator) on the x-axis and the monitor (Hyfe) on the y-axis. Points are intentionally jittered by up to 0.3 values so as to provide more visibility on high density areas. The diagonal line (slope = 1, intercept = 0) represents where each point would fall in the hypothetical case of a perfect monitor.

In three recording sessions, Hyfe had a sensitivity around 55%: sessions 2, 17 and 38 ( Figure 3). These sessions met the quality criteria of more than 10 sounds unanimously classified as coughs. Potential explanations for this performance include the acoustic characteristics of the solicited coughs from these particular participants and the level of background noise. Coughs in session 2 and 17 had uncommon acoustic characteristics, such as biphasic decibel peaks, and different spectrographic features. Session 38 had significantly more background noise than the others. Sensitivity for the Session 20 was not evaluated because this was a patient with refractory chronic cough that generated hundreds of out-of-script, making timestamping impossible. We found the Pearson correlation of Hyfe to the gold standard to be 0.968 ( Figure 4).

### Limitations

The major limitation was that this study of performance evaluation was done in a laboratory “in-vitro” environment, not community or a clinical setting. During this study, phone microphones were oriented towards and phones were placed at 50 cm from the participant, however, these settings would vary in real life clinical scenarios with coughing patients which could have longer distances and obstructing objects in between.

## Discussion

The ability to unobtrusively monitor cough has the potential to greatly improve patient care, public health and drug development. The uptake of cough monitoring technologies will be determined by their usability, their clinical performance and the increasing evidence that they can provide actionable information for clinical decision making. Hyfe has advantages over existing cough monitors as it can run in the background of a smartphone and passively monitor coughs for longer than 24h of recordings. Rather than using special equipment and limited time windows for continuous cough monitoring, the use of this novel system improves the efficiency of monitoring and reduces the monitoring costs.

There are many ways to assess cough detectors accuracy. The intrinsic, or analytical, performance of AI-based cough monitors directly results from their algorithm’s sensitivity and specificity for labeling recorded sounds. However, those same monitoring technologies may perform differently when deployed in various clinical settings where the acquisition of such sounds may represent a challenge in the first place, leading to either unrecorded coughs or recorded and misclassified non-cough sounds. We previously reported on the analytical performance of Hyfe. ^13^ Here we report on its pre-clinical performance using scripted solicited coughs in a controlled environment.

Defining a gold standard for the performance evaluation of passive cough monitors represents a challenge which we addressed with standardized procedures ensuring human listener inter-observer consensus. This process and our results highlight three important issues related to evaluating cough monitors. Firstly, it is critical to have a precise method of aligning different data streams. Our failure to have this resulted in the exclusion of two sessions. Going forward, we propose the use of a distinct auditory signal, or “coda”, that can be played at the beginning of each session so both the continuous audio recorder and the smartphone running the app will have a series of characteristic peak sounds that can be used for timestamping and alignment. The coda currently used for Hyfe-related studies is available on YouTube. Secondly, although solicited coughs have been used to validate cough-counting devices in the past ^20^ and previous literature reports that spontaneous and solicited coughs have similar acoustic characteristics, ^21^ we found significant differences in the sound of solicited coughs from different study participants. When asked to voluntarily cough, ten of the 49 research subjects generated sounds that were not unanimously recognized as coughs by human annotators. This observation raises questions about the utility of solicited coughs for diagnostic purposes. Finally, there are interpersonal differences in how sounds are classified by annotators. Because of this we had to exclude 88 sounds from the analysis. This has prompted additional efforts to minimize interobserver variability by developing clear operating procedures and training programs for cough annotators. We propose that protocols such as these be shared, and that consensus be sought so as to facilitate comparison of monitoring technology.

**Figure 5.**
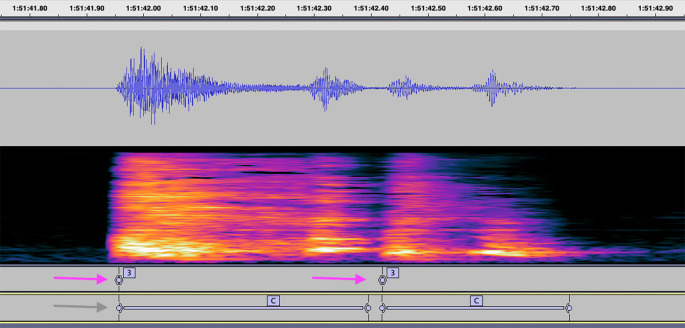
Example labels indicating cough placed by a human listener in a single second. Purple arrows indicate labels placed in this study, according to the 4-tier SOP. The gray arrow indicates how this audio segment would be annotated using the updated 6-tier SOP.

After this study, we believe that labeling cough duration rather than just its beginning has more value in further training Hyfe’s AI model, also in analyzing agreement between human listeners, and agreement with Hyfe ( Figure 5). Therefore, the updated 6-tier version SOP was proposed, which is currently being used for cough labeling in continuous audio recordings.

Environmental sounds may interfere with capturing coughs in real life, as seen in the sensitivity of session 38 ( Figure 3), however, continuous improvements of the AI peak detection models and the cough classifiers, may address this potential issue in the near future. Even though we have not observed any significant differences in the quality of smartphones used in this trial, there might be cases when the version of smartphone operating system plays some role in smartphone’s general usability and experience for the user.

Overcoming these challenges, we were able to evaluate Hyfe’s accuracy using 1679 solicited sounds generated by a total of 37 subjects. Hyfe’s overall sensitivity and specificity were respectively 91% and 98% and did not differ significantly between two phones. Importantly, we feel the more relevant parameter of performance to be the Pearson correlation of the cough rates as measured by the device and the gold standard (human annotation), which was 0.968. We propose that going forward, analysis of cough monitors should use correlation in rates (gold standard vs monitor detections) as the primary metric of their performance. Though we used a minute (due to the highly condensed nature of the study), in most continuous monitoring use cases, coughs per *hour* is likely to be the most clear and useful period of observation.

Of note, the performance was lower in four subjects, presumably due to the intrinsic acoustic characteristics of solicited coughs and the level of background noise.

Further validation studies will need to be conducted in the specific clinical settings in which Hyfe is intended to be used. To better contextualize and design such trials, target product performance specifications will be required and are expected to differ significantly between use cases. Lessons can be learned from other types of monitors such as fitness trackers, whose results can differ from each other by up to 30%. ^22^ Whereas, regulated medical devices used in clinical practice will require greater precision. The presented data here is encouraging, suggesting that Hyfe’s performance is adequate to be used in most clinical and research contexts. Taken together, these results show that AI-enabled systems might provide a valuable tool for objectively, and unobtrusively monitoring cough.

## Data availability

### Underlying data

Github: ericmkeen/navarra: Hyfe performance evaluation, https://doi.org/10.5281/zenodo.6710562. ^23^

This project contains the following underlying data:

• 01.results. R (takes pre-formatted datasets and carries out performance evaluation, plots results)
• detections.csv (Hyfe detections data)
• hyfe_performance.R (analysis of Hyfe performance)
• labels.csv (human labeled data)
• offsets_emk.csv (automatic and manual offsets made to the data)

## Software

Software available from:

R version 2.04 (RStudio Team, 2020), available from https://cran.r-project.org/bin/windows/base/old/4.0.2/

Hyfe, version acl 1.24.4, available from https://www.hyfe.ai/

Audacity | Free, open source, cross-platform audio software for multi-track recording and editing, available from https://www.audacityteam.org/
