# Supplementary material for: Performance evaluation of the smartphone-based AI cough monitoring app - Hyfe Cough Tracker against solicited respiratory sounds
Source: F1000Res. 2023 Jun 9;11:730. Originally published 2022 Jul 1. [Version 2] doi: 10.12688/f1000research.122597.2 (PMC11809693; doi:10.12688/f1000research.122597.2)
Supplement: Peer review history

# Reviewer report: Reviewer response for version 2

**DOI:** 10.5256/f1000research.147557.r351386

**Published:** 2025-01-07

**Authors:** Richard D Turner

**Affiliations:** 1 Respiratory Medicine, Princess Alexandra Hospital (Ringgold ID: 1966), Woolloongabba, Queensland, Australia; 2 Griffith University, Southport,, QLD, Australia

The authors are to be commended for openly sharing their data on an aspect of validation work of their Hyfe cough detection algorithm. The paper has already undergone a quite extensive review process by two other reviewers, and changes to the manuscript made. I have no major comments to make. This is only part of the validation work necessary, as is made clear by the authors. Assessing the detection of cough sounds by testing the algorithm on volitional noises made by healthy volunteers has limitations, but this is pointed out in the manuscript. Assessing sensitivity and specificity of automated cough detection also clearly needs to be done with patients, in the context of both low and high cough frequency, and in differing acoustic environments, with vary degrees of background noise, including with the presence of other subjects who are also coughing. I believe a lot of this work is being or has already been done by this study group.

 I just have some minor comments which are as follows.

 Introduction

 Check reference 6 halfway through the first paragraph – I would suggest a more relevant citation for cough patient-reported outcomes.

 Results

 Paragraph 3, first sentence: “In three recording sessions…”. I would suggest writing as “In three of the 37 recording sessions evaluated…”. This emphasises that the sensitivity was low in only the minority of situations.

 Figure 6 does not currently show a standard Bland-Altmann plot, as stated by another reviewer. The axes should be corrected as previously described.

 Discussion

 Paragraph 3. The link to the YouTube page containing the “coda” for aligning Hyfe recordings does not work without a password – please change, or explain.

Is the work clearly and accurately presented and does it cite the current literature?

Yes

If applicable, is the statistical analysis and its interpretation appropriate?

Partly

Are all the source data underlying the results available to ensure full reproducibility?

Yes

Is the study design appropriate and is the work technically sound?

Yes

Are the conclusions drawn adequately supported by the results?

Yes

Are sufficient details of methods and analysis provided to allow replication by others?

Yes

Reviewer Expertise:

Clinical chronic cough, including measurement and treatment.

I confirm that I have read this submission and believe that I have an appropriate level of expertise to confirm that it is of an acceptable scientific standard.

# Reviewer report: Reviewer response for version 2

**DOI:** 10.5256/f1000research.147557.r276549

**Published:** 2024-05-14

**Authors:** Terence E. Taylor

**Affiliations:** 1 Vitalograph Ireland Ltd., Ennis, Ireland

The manuscript presents a basic proof-of-concept study of the Hyfe system on a limited dataset using a highly constrained experimental setup, therefore, limiting the interpretation of the performance evaluation of the Hyfe system even within a controlled environment. Some of my previous comments have been addressed by the authors. However, a significant number of my suggested additions, which could have greatly improved the manuscript, in my opinion, were mostly not considered by the authors. So, it is clear the authors will not consider further analyses/experimentation that in my view are most definitely within the scope of evaluating the performance of the Hyfe system in a controlled environment. Acknowledging all of this, I will update my decision to Approved With Reservations pending the authors consider the comments listed below.

 Comments:

 The title must reflect this being a basic proof-of-concept study. A suggested title is “A smartphone-based AI cough monitoring app - Hyfe Cough Tracker: A proof-of-concept study using solicited respiratory sounds”. The authors need to change wording relating to “performance evaluation” of the system throughout the manuscript to proof-of-concept study.

 Please report algorithm results on total coughs per subject. Even given the authors’ suggestion that a correlation may exist between total coughs and cough seconds, the manuscript should still report the results on detecting all coughs.

 Is Figure 4 showing the number of cough-seconds per minute or number of coughs per minute?

 Thank you for adding Figure 5. The data point at the top right corner seems to be heavily influencing the regression lines. Is it an outlier? If so, please remove it and re-plot the regression line and report the correlation values without it. If that data point is related to a subject with many coughs, it would be of interest to briefly note in the Discussion the algorithm performance of that individual subject’s data if it is to be removed from the statistical analyses.

 Thank you for adding Figure 6. However, I think there was a misinterpretation here. To clarify, the Bland-Altman plot should consist of the mean of the two measurements on the x-axis with the difference between the two measurements on the y-axis (Hyfe-Annotator). Please regenerate this plot. The request for the absolute percentage error in the first review was a separate metric to report, not related to the Bland-Altman. Please additionally report the overall mean absolute percentage error in terms of total coughs and cough-seconds across all subjects.

 I acknowledge the authors have removed suggestions that Hyfe can be used in most clinical settings based on the results of this study. However, the following newly added sentence should also be removed: “The presented data here is encouraging, suggesting that Hyfe’s performance is adequate to proceed to validation in clinical context”. I appreciate the authors have clinical studies that they are working on, but the limited scope of this proof-of-concept study does not back up this statement, hence, it should be removed.

 Lastly, my affiliation to Vitalograph (Ireland) Ltd. has always been disclosed on my review for this manuscript. The editorial team requested that I reiterate this in the competing interest section, which I have done, and this is published. Having vast academic and industrial experience in the field of audio signal processing for respiratory applications, my review of this manuscript is solely based on scientific merit and contribution to the field. The authors I hope can appreciate the significant time taken to review their manuscript, having provided detailed feedback to enhance it.

Is the work clearly and accurately presented and does it cite the current literature?

No

If applicable, is the statistical analysis and its interpretation appropriate?

Partly

Are all the source data underlying the results available to ensure full reproducibility?

No

Is the study design appropriate and is the work technically sound?

No

Are the conclusions drawn adequately supported by the results?

Partly

Are sufficient details of methods and analysis provided to allow replication by others?

No

Reviewer Expertise:

audio signal processing, cough sound analysis, artificial intelligence, telehealth, respiratory diseases

I confirm that I have read this submission and believe that I have an appropriate level of expertise to confirm that it is of an acceptable scientific standard, however I have significant reservations, as outlined above.

# Reviewer report: Reviewer response for version 2

**DOI:** 10.5256/f1000research.147557.r177694

**Published:** 2024-05-10

**Authors:** Terence E. Taylor

**Affiliations:** 1 Vitalograph Ireland Ltd., Ennis, Ireland

The manuscript presents a basic proof-of-concept study of the Hyfe system on a limited dataset using a highly constrained experimental setup, therefore, limiting the interpretation of the performance evaluation of the Hyfe system even within a controlled environment. Some of my previous comments have been addressed by the authors. However, a significant number of my suggested additions, which could have greatly improved the manuscript, in my opinion, were mostly not considered by the authors. So, it is clear the authors will not consider further analyses/experimentation that in my view are most definitely within the scope of evaluating the performance of the Hyfe system in a controlled environment. Acknowledging all of this, I will update my decision to Approved With Reservations pending the authors consider the comments listed below.

**Comments:**

 The title must reflect this being a basic proof-of-concept study. A suggested title is “A smartphonebased AI cough monitoring app - Hyfe Cough Tracker: A proof-of-concept study using solicited respiratory sounds”. The authors need to change wording relating to “performance evaluation” of the system throughout the manuscript to proof-of-concept study.

 Please report algorithm results on total coughs per subject. Even given the authors’ suggestion that a correlation may exist between total coughs and cough seconds, the manuscript should still report the results on detecting all coughs.

 Is Figure 4 showing the number of cough-seconds per minute or number of coughs per minute?

 Thank you for adding Figure 5. The data point at the top right corner seems to be heavily influencing the regression lines. Is it an outlier? If so, please remove it and re-plot the regression line and report the correlation values without it. If that data point is related to a subject with many coughs, it would be of interest to briefly note in the Discussion the algorithm performance of that individual subject’s data if it is to be removed from the statistical analyses.

 Thank you for adding Figure 6. However, I think there was a misinterpretation here. To clarify, the Bland-Altman plot should consist of the mean of the two measurements on the x-axis with the difference between the two measurements on the y-axis (Hyfe-Annotator). Please regenerate this plot. The request for the absolute percentage error in the first review was a separate metric to report, not related to the Bland-Altman. Please additionally report the overall mean absolute percentage error in terms of total coughs and cough-seconds across all subjects.

 I acknowledge the authors have removed suggestions that Hyfe can be used in most clinical settings based on the results of this study. However, the following newly added sentence should also be removed: “The presented data here is encouraging, suggesting that Hyfe’s performance is adequate to proceed to validation in clinical context”. I appreciate the authors have clinical studies that they are working on, but the limited scope of this proof-of-concept study does not back up this statement, hence, it should be removed.

 Lastly, my affiliation to Vitalograph (Ireland) Ltd. has always been disclosed on my review for this manuscript. The editorial team requested that I reiterate this in the competing interest section, which I have done, and this is published. Having vast academic and industrial experience in the field of audio signal processing for respiratory applications, my review of this manuscript is solely based on scientific merit and contribution to the field. The authors I hope can appreciate the significant time taken to review their manuscript, having provided detailed feedback to enhance it.

Is the work clearly and accurately presented and does it cite the current literature?

No

If applicable, is the statistical analysis and its interpretation appropriate?

Partly

Are all the source data underlying the results available to ensure full reproducibility?

No

Is the study design appropriate and is the work technically sound?

No

Are the conclusions drawn adequately supported by the results?

Partly

Are sufficient details of methods and analysis provided to allow replication by others?

No

Reviewer Expertise:

audio signal processing, cough sound analysis, artificial intelligence, telehealth, respiratory diseases

I confirm that I have read this submission and believe that I have an appropriate level of expertise to confirm that it is of an acceptable scientific standard, however I have significant reservations, as outlined above.

# Reviewer report: Reviewer response for version 1

**DOI:** 10.5256/f1000research.134609.r148456

**Published:** 2022-09-08

**Authors:** Terence E. Taylor

**Affiliations:** 1 Vitalograph Ireland Ltd., Ennis, Ireland

**Editorial note [4th May 2023]:**

 A potential conflict of interest has come to light and so we have added this to the report to ensure full transparency.

 The aim of the Hyfe app is to provide a portable and high performing system for measuring cough rate. The authors describe an evaluation of the Hyfe app using 10-minute audio recordings consisting of cough and speech obtained from participants in a controlled environment. Audio data were collected using two identical smartphones and an additional MP3 recorder placed 50cm to participants.

 While I find this area of research quite interesting, I have several major concerns with this study from the data collection to how the evaluation was measured. The type and amount of data does not reflect how the app would be used in real-life settings. In my opinion, this study does not provide sufficient evidence that the Hyfe system can perform adequately in real-life clinical research, particularly over longer periods of time (24 hours or above). Furthermore, I believe this study lacks novelty compared to other audio-based cough detection studies and represents more of a pilot study than an evaluation of the system. Please see further comments below.

**Abstract:**

- In Objectives, please change the relevant sentence to “We evaluate performance of cough detection using continuous audio recordings obtained within a controlled environment and cough counting by trained individuals as the gold standard.”

**Introduction:**

- MAJOR: In ref 13, it is noted that participants in this other study complained of increased battery consumption. And so, the evaluation there was constrained to 6-hour windows. Has this app been ever evaluated over at least 24-hour periods? According to the authors, one of the advantages of Hyfe is the ability to passively record for extended periods of time (i.e. greater than 24 hours). Has there been analysis done on the battery consumption effects over longer periods? In my opinion, this study should have monitored cough over at least 24 hours to show a more realistic evaluation of the system.
- MINOR: Privacy concerns are mentioned in the introduction. How does Hyfe preserve privacy exactly? If this information has already been published elsewhere, please cite the relevant literature and give a brief explanation on how privacy preservation is achieved.

**Methods:**

- MINOR: What versions of iOS/Android has the Hyfe app been tested on?
- MINOR: Why were baseline respiratory symptoms not considered for participants? Cough sounds can vary in terms of acoustic properties across different respiratory disease types. The data reported here have no mention of being evaluated on any respiratory disease cough sounds.
- MINOR: Regarding the CNN model, was it trained on sufficient data across different smartphones, microphone locations, disease types? Please comment on this.
- MAJOR: Why place the smartphone 50cm away from the participant? What is the approximate distance between a user’s mouth and a smartphone mic when they are interacting with their smartphone in front of them with smartphone-in-hand? Was this thought of to replicate real-life use? I would have thought that in many real-life cases also, a lot of the audio that Hyfe would capture would be when the smartphone is located inside the user’s pocket or bag. Why not place one smartphone in the participant’s pocket and have one in their hand to try replicate real-life use? Why constrain the recording protocol so heavily? Also, why use two of the same identical smartphones? It would have been useful to compare different smartphones with different builds, battery consumption properties, microphone designs etc. Was the gain/dynamic range of the microphones taken into account? All these points significantly limit the interpretability of this evaluation in relation to how Hyfe could perform in clinical settings in my opinion. The data collection is not representative of real-life use.
- MINOR: What is the sampling rate and bit depth of the audio recordings? Please report this.
- MINOR: It would be useful to have an English language version of the pre-generated computer script also available to the reader.
- MINOR: How were participants instructed to cough exactly? Was it for a certain amount of time? For a certain number of explosive cough sounds? Please explain briefly.
- MAJOR: Recording sessions occurred in a quiet room as reported. What does “quiet” mean here? Were there sound intensity levels recorded? Was the room acoustically insulated? What were the signal-to-noise ratio (SNR) values of the different sessions? Please provide this information to help the reader understand the environment the recordings were obtained in.
- MAJOR: What were the kappa agreement scores to show inter-rater agreement for the manual labeling? Why do you need a 4-tier labeling system when users were prompted to perform specific sounds? Do you not already know when a user coughed as they were prompted to do so at specific points within the 10min session? I understand the prompts were randomized, but was this not recorded internally for post-analysis? Therefore, why is there a need for labels 1 and 2 if you have a log of what the participants were doing during each part of the recordings?
- MINOR: How many sounds were labeled 0, 1, 2 and 3? Please report this information. How many explosive cough sounds on average were there in each cough audio segment? Were participants prompted to perform a certain number of explosive cough sounds?
- MINOR: Why does the Hyfe system classify every 0.5 seconds, but labeling was done every 1 second?
- MAJOR: Multiple coughs may occur within a 1 second window. Why label a full 1 second window as a single true positive cough even if there could be multiple single manually labeled coughs within the 1 second? In my opinion, cough rate estimation would be more accurate if all coughs were considered rather than labeling a 1 second window of coughing as a single cough event (when estimating app performance). Is there reasoning for this in the literature? If so, please add relevant citations. If I understand correctly, it means technically you could miss all but one cough in a given 1sec window and still have very high sensitivity/specificity based on your labeling system? If this is the case, this is a major limitation. How coughs are grouped together in peals or bouts can have a significant impact on cough rate estimation. I think this discrepancy would become more evident had the system been evaluated on a cough-by-cough basis and over longer periods on patients with respiratory disease as they may tend to cough in groups in quick succession (within 1 second in many cases).
- MINOR: Was there any overlap in the labeling of 1 second windows i.e. 50% overlap between consecutive windows for labeling?
- MAJOR: Sample Size: “385 sounds” – are you referring to cough sounds here, or a mix of both cough and non-cough? How did you determine the balance of data between cough and non-cough (speech, throat clear, noise etc.)? It seems a balance of approximately 1:2 (coughs to non-coughs) was employed. In real-life settings, over the course of a 24-hour period for example, the number of non-cough segments will far outweigh the number of cough segments if Hyfe was to continuously record audio. This does not seem to be considered here. If the data were to be more realistically balanced in this way, it may have a significant effect on the positive predictive value (PPV) of the algorithm (PPV needs to be reported). One of the biggest challenges of longer term (24 hours or more) audio-based cough monitoring is the PPV performance measure.
- MAJOR: One of the advantages of Hyfe, reported by the authors, is that it can monitor cough rate over longer periods (>24 hours). But this study looked at 10-minute recording windows. Even taking into account that no respiratory symptoms were not noted from participants (which I think is a major limitation), I think the balance of data here are not truly representative of real-life settings especially if the authors suggest Hyfe can monitor cough rate over 24-hour periods. This is a major limitation to this evaluation study. This system needs to be evaluated over at least 24-hour periods before it can be suggested that it may perform strongly for longer term analysis in clinical settings.
- MINOR: Synchronization of labels - Please provide a clearer explanation for this. Why synchronize to within 2 seconds? This seems like a long duration considering multiple explosive cough sounds may occur within 2 seconds.
- MINOR: Data were discarded if they had less than 10 cough seconds of agreement. How often did that occur? How much data were discarded overall? – from Figure 1 it seems 20-25% of data were discarded due to inadequate quality. How would this reflect real-life evaluation then? The app would be unsure of about 20% of coughs at best? That seems to be a potential large margin of error. Please comment on this.
- MAJOR: Was the 4-tier or 6-tier labeling SOP used? This is quite confusing for the reader. If 6-tier is superior in the opinion of the authors, why not revert and apply it to all data and re-run the analysis? I understand it may mean re-training the CNN but if it will be beneficial as mentioned by the others, then it should be reported here.
- MINOR: “Because the utility of a cough monitor is not in noting individual coughs but rather in tracking cough rates…”. Surely identifying singular coughs should be the goal to obtain the most accurate estimation of cough rate? If not, you could hypothetically be detecting false positives and false negatives, but still hit the correct cough rate. This could affect the analysis of how cough rate changes over time throughout a 24-hour period, for example, if it were of interest. Please provide a stronger argument for this.

**Results: **

- MAJOR: Please report SNR for audio sessions in comparison to cough. It may help the reader understand the poor performance of sessions 2, 17 and 38. Real-life recordings will be full of different types and levels of noise. If it is only possible to evaluate on audio within controlled environments, then the authors should augment the data by adding various different types and levels of noise to the data to replicate real-life use.
- MINOR: What were the uncommon acoustic characteristics associated with Sessions 2 and 17? An additional figure with time domain plot as well as a spectrogram plot of coughs from these participants would be useful here for the reader to understand why the system did not perform as well for these participants.
- MINOR: Figure 3 image quality should be improved.
- MINOR: “Sensitivity for the Session 20 was not evaluated because this was a patient with refractory chronic cough that generated hundreds of out-of-script, making timestamping impossible.” – Please elaborate further. Were there too many coughs? One would assume the data from this participant would have been very interesting to analyze and run through the Hyfe system?
- MINOR: Limitations: I agree with the limitations mentioned. So why evaluate the Hyfe system in a way it will rarely if not never be used in? Please comment on this.
- MAJOR: Please give details of the effect the Hyfe app has on battery life. One would think a continuous audio recording will significantly drain a smartphone battery. It seems to be mentioned in a previous study (ref 13). Were there experiments done on this? If not, this analysis needs to be performed and reported in this evaluation study.
- MAJOR: A Bland Altman plot analyzing cough rate between Hyfe and manual labels is required here. Correlation analysis is not enough to convince the reader that the Hyfe system can accurately estimate cough rate (unless you were to use the linear regression equation to map Hyfe estimation to manual estimation which I don’t think is the goal here). While strong correlation is interesting to observe, simply showing the equal line on Figure 4 does not highlight if there is any bias in cough rate estimation. Please add the linear regression line and equation in Figure 4. Please also add an additional panel for a Bland Altman plot comparing the mean cough rate between manual and Hyfe to the difference in cough rate between manual and Hyfe.

**Discussion:**

- MAJOR: Where does the score threshold of 0.85 come from? Can you report the distribution of CNN output probability scores for all label categories as supplementary material? A histogram, for example, of probability scores for each label category would be interesting to see and could enhance the reader’s understanding of the challenges in labeling category 1 and 2 sounds.
- MINOR: The discussion on inter-participant variability and the debate on using solicitated voluntary coughs as a diagnostic tool is quite interesting. Does this suggest these are most likely voluntary throat clears rather than involuntary coughs? As a result, why then prompt the user to record a voluntary cough when first using the app?
- MINOR: What difference will the 6-tier SOP make for training the CNN model? I assume fixed 0.5sec segments are employed for training the model so how will the duration markers be more beneficial than just the onset markers used in the 4-tier SOP labeling system?
- MAJOR: The Pearson correlation is interesting, but I disagree that it should be the primary metric of performance. I think the authors should perform a linear regression analysis also to analyze the slope of the regression line. Only using Pearson correlation does not disclose information regarding how many coughs are missed in detection. For example, you could theoretically have a Pearson coefficient of 1 but you are missing 1 cough in every 2 or 3 coughs consistently in your detection model (this would be reflected in the slope of the regression analysis). The Pearson correlation metric is one of a group of metrics that should be considered in my opinion. I would like to see linear regression analysis, absolute percentage error according to the total number of coughs per participant and a Bland Altman analysis.
- MAJOR: “…Hyfe’s performance is adequate to be used in most clinical and research contexts.” This study does not suggest this in my opinion if the data were collected in controlled environments, was not evaluated on real respiratory patients and the smartphones were not evaluated in multiple different positions/locations.

**Other Comments**:

- MAJOR: While I understand the data were obtained in controlled environments, in my opinion, more data are required to obtain a more realistic measure of performance in order to suggest Hyfe could be used in clinical settings. This includes more data using different smartphone locations (in user’s pocket, bag etc.), more realistic data with different types and levels of noise (including muffled coughs due to hand/mask covering) and I think it should be evaluated on a cough-by-cough basis rather 1 second windows as a form of cough rate.
- MINOR: How does the app know if it is the user who coughed and not a person beside them in a crowded area? Has this been taken into account within the CNN training data? Please comment on this.
- MINOR: Does the Hyfe app still record audio when the user is on a phone call or sending voice messages?
- MINOR: Does the app work without internet connectivity?
- MINOR: SOP4 Supplementary Document: Page 4, Section 3, bullet 3 – should that read as “…at the end of the expulsive phase..”?
- MINOR: SOP4 Supplementary Document: I think reporting a cough as having one sound or two sounds is a little confusing. Consider moving Fig. 5 and the paragraph above it to before Fig. 3. Start with showing the sound, explaining the three phases of the cough sound event and then describe in more detail the physiology behind it. Fig. 3 panel B needs to highlight that the voiced phase is missing. It is a little confusing to just say A has a “double” sound and B has a “single sound”.
- MAJOR: SOP4 Supplementary Document: Page 8, Table 1 – according to the 4-tier process, the label 2 may be due to the phone located in the user’s pocket which, I assume, will be a vastly common occurrence if this app were to be used in cough clinical applications. In this study label 2 coughs weren’t considered in the app evaluation. Why not? I think it is essential particularly for an app-based cough monitoring system to be evaluated on recordings when the phone is in the user’s pocket or bag for example.
- MINOR: SOP4 Supplementary Document: Page 8, Labeling Tips – I think the label 2 may highlight a potential limitation of the Hyfe system. It is indicating to the reader that the smartphone needs to be in close proximity to the user. Coughs may sound “distant” even if the user is coughing. Please elaborate what “distant” means here. Is this related to the audio amplitude of the cough sound? Certain types of coughs may have lower amplitudes, particularly if a very ill patient has exhausted their respiratory strength.
- MAJOR: The smartphone can be located on a table with many other people in the user’s vicinity. How do you know it is the user’s cough? This could be a major limitation. Particularly if the intention is to detect subtle changes in cough rate. There are many external factors when using an app-based audio cough detection system that can affect cough rate estimation. I don’t think the authors make it clear how they will evaluate this or can overcome such challenges using this system.
- MINOR: If possible, it would be very beneficial to have examples of the sounds that were recorded in the controlled environment for readers to listen to.

Is the work clearly and accurately presented and does it cite the current literature?

No

If applicable, is the statistical analysis and its interpretation appropriate?

Partly

Are all the source data underlying the results available to ensure full reproducibility?

No

Is the study design appropriate and is the work technically sound?

No

Are the conclusions drawn adequately supported by the results?

Partly

Are sufficient details of methods and analysis provided to allow replication by others?

No

Reviewer Expertise:

audio signal processing, cough sound analysis, artificial intelligence, telehealth, respiratory diseases

I confirm that I have read this submission and believe that I have an appropriate level of expertise to state that I do not consider it to be of an acceptable scientific standard, for reasons outlined above.

# Response

**Published:** 2023-04-27

**Authors:** Mindaugas Galvosas

**2.1**

**Comment: **While I find this area of research quite interesting, I have several major concerns with this study from the data collection to how the evaluation was measured. The type and amount of data does not reflect how the app would be used in real-life settings. In my opinion, this study does not provide sufficient evidence that the Hyfe system can perform adequately in real-life clinical research, particularly over longer periods of time (24 hours or above). Furthermore, I believe this study lacks novelty compared to other audio-based cough detection studies and represents more of a pilot study than an evaluation of the system. Please see further comments below.

**Answer: **Firstly we want to raise a serious issue with this review. Terence Taylor is a Senior Clinical Data Scientist working for Vitalograph Ireland Ltd, a for profit company that manufactures the Vitalojak, a device used to quantify cough in 24 hour windows in the context of clinical trials. This product could be affected by the emergence of new technologies capable of monitoring cough for longer periods of time. There is a clear economic conflict of interest and the reviewer should have either recused himself or clearly disclosed it. None of these potential remedial actions were taken.  

 We find it equally appalling that the editorial team of F1000 research has failed to act on this after the issue was raised.

 Here we provide point by point answers to the reviewer´s comments assuming good will, even when the repeated demands for experiments and data outside the scope of this manuscript clearly suggest otherwise.

 This was the laboratory evaluation of our algorithm, and this manuscript is not meant to demonstrate how it performs in real world environments. Such evaluation, which is a necessary next step, is being done and will be provided as a separate manuscript showing real-world validation of the technology. The scope of this manuscript did not include real-life scenarios and long-term monitoring.

 The novelty of this study is purely in evaluating the performance of novel fully automated sound capture, cough detection and cough quantification system.

**Actions: **None taken.

**2.2**

**Comment:**

 Abstract: In Objectives, please change the relevant sentence to “We evaluate performance of cough detection using continuous audio recordings obtained within a controlled environment and cough counting by trained individuals as the gold standard.”

**Answer: **Abstract edited as per reviewer recommendation.

**Actions: **Abstract edited as per reviewer recommendation.

**2.3**

**Comment: **Introduction: MAJOR: In ref 13, it is noted that participants in this other study complained of increased battery consumption. And so, the evaluation there was constrained to 6-hour windows. Has this app been ever evaluated over at least 24-hour periods? According to the authors, one of the advantages of Hyfe is the ability to passively record for extended periods of time (i.e. greater than 24 hours). Has there been analysis done on the battery consumption effects over longer periods? In my opinion, this study should have monitored cough over at least 24 hours to show a more realistic evaluation of the system

**Answer: **This question is not related to the experiment described in the manuscript, which was purely evaluating the performance of the algorithm. “…one of the advantages of Hyfe is the ability to passively record for extended periods of time (i.e. greater than 24 hours).” – we have conducted separate works that followed-up for long periods of time (months) and that is available: (reference 12, doi: 10.1183/23120541.00001-2022; also reference 14, https://doi.org/10.1183/23120541.00053-2022 )

**Actions: **References of long-term monitoring are reflected (12, 14).

**2.4**

**Comment: **Introduction: MINOR: Privacy concerns are mentioned in the introduction. How does Hyfe preserve privacy exactly? If this information has already been published elsewhere, please cite the relevant literature and give a brief explanation on how privacy preservation is achieved.

**Answer: **Privacy concerns are described in previous publications (references of this manuscript 12-14) that include real patients and monitoring in real world environments. The scope of this manuscript was not to overview the whole monitoring process, but to evaluate the performance of the technology in controlled environments with solicited coughs.

**Actions: **Manuscript references 12-14 address privacy concerns and describe privacy preserving in detail.

**2.5**

**Comment: **Methods: MINOR: What versions of iOS/Android has the Hyfe app been tested on?

**Answer: **This study was done with Android phones only and all used Android version 11.

**Actions: **Android version 11 is mentioned in the manuscript.

**2.6**

**Comment: **Methods: MINOR: Why were baseline respiratory symptoms not considered for participants? Cough sounds can vary in terms of acoustic properties across different respiratory disease types. The data reported here have no mention of being evaluated on any respiratory disease cough sounds.

**Answer: **Here again, the objective of this work was to evaluate the performance on solicited coughs. There is ongoing work validating the algorithm in clinical patients in real-life environments.

**Actions: **None taken.

**2.7**

**Comment: **Methods: MINOR: Regarding the CNN model, was it trained on sufficient data across different smartphones, microphone locations, disease types? Please comment on this.

**Answer: **It was done with a broad range of microphones and collected from various locations and it has been added to the manuscript.

**Actions: **Added to “Methods”: “Hyfe’s CNN model, at the time of this analysis, was trained on more than 200M real-world cough and cough-like samples, collected from multiple countries and multiple devices.”

**2.8**

**Comment: **Methods: MAJOR: Why place the smartphone 50cm away from the participant? What is the approximate distance between a user’s mouth and a smartphone mic when they are interacting with their smartphone in front of them with smartphone-in-hand? Was this thought of to replicate real-life use? I would have thought that in many real-life cases also, a lot of the audio that Hyfe would capture would be when the smartphone is located inside the user’s pocket or bag. Why not place one smartphone in the participant’s pocket and have one in their hand to try replicate real-life use? Why constrain the recording protocol so heavily? Also, why use two of the same identical smartphones? It would have been useful to compare different smartphones with different builds, battery consumption properties, microphone designs etc. Was the gain/dynamic range of the microphones taken into account? All these points significantly limit the interpretability of this evaluation in relation to how Hyfe could perform in clinical settings in my opinion. The data collection is not representative of real-life use.

**Answer: **Here again, you are requesting information which goes beyond the scope of the work described in the manuscript. The main objective of this work was to evaluate the performance (sound capture, cough detection and quantification) of the AI model on solicited coughs in a controlled environment.

**Actions: **None taken.

**2.9**

**Comment: **Methods: MINOR: What is the sampling rate and bit depth of the audio recordings? Please report this.

**Answer: **The sampling rate was 44.1 Hz and the files are 16-bit.

**Actions: **Added to study design: “The sampling rate was 44.1 Hz and the files are 16-bit.”

**2.10**

**Comment: **Methods: MINOR: It would be useful to have an English language version of the pre-generated computer script also available to the reader.

**Answer: **We fail to see the additional value of a translation, given that the Spanish words were chosen based on their pronunciation and cough-like sounding.

**Actions: **None taken.

**2.11**

**Comment: **Methods: MINOR: How were participants instructed to cough exactly? Was it for a certain amount of time? For a certain number of explosive cough sounds? Please explain briefly.

**Answer:** This is a valid comment. Participants were instructed to cough once every time they were prompted by the script to do so.

**Actions: **Added “Participants were instructed to cough once every time they were prompted by the script to do so.”  to “Study design”.

**2.12**

**Comment: **Methods: MAJOR: Recording sessions occurred in a quiet room as reported. What does “quiet” mean here? Were there sound intensity levels recorded? Was the room acoustically insulated? What were the signal-to-noise ratio (SNR) values of the different sessions? Please provide this information to help the reader understand the environment the recordings were obtained in.

**Answer: **This is a valid comment. Sound intensity levels were monitored using https://meters.uni-trend.com/product/ut353-ut353bt/ and never exceeded 110dB.

 The room was not acoustically insulated.

**Actions: **Added to the “Study design”: “Sound intensity levels in the room were also monitored using UNI-T mini sound level meter. The room was not acoustically insulated”.

 Added to the “Results”: “Sound levels in the room during the study were never above 110dB.”

**2.13**

**Comment: **Methods: MAJOR: What were the kappa agreement scores to show inter-rater agreement for the manual labeling? Why do you need a 4-tier labeling system when users were prompted to perform specific sounds? Do you not already know when a user coughed as they were prompted to do so at specific points within the 10min session? I understand the prompts were randomized, but was this not recorded internally for post-analysis? Therefore, why is there a need for labels 1 and 2 if you have a log of what the participants were doing during each part of the recordings?

**Answer: **The sounds were indeed randomized and triple-human labeling was used as the gold standard. The nature of an elicited sound, even though the instruction is to produce a cough, varies depending on the person (some may get confused and do throat clears when asked to cough, etc).

 We agree that inter-rater agreement is an interesting point to look at. There is a separate full paper in preparation looking specifically at this with a whole different dataset.

**Actions:** Added  a linear analysis plot and Bland-Altman plot in the “Results” section.

**2.14**

**Comment: **Methods: MINOR: How many sounds were labeled 0, 1, 2 and 3? Please report this information. How many explosive cough sounds on average were there in each cough audio segment? Were participants prompted to perform a certain number of explosive cough sounds?

**Answer: t**he dataset has been made public with this manuscript. The number of sounds instructed was the same for all participants, as prompted by the script.

**Actions: **Added to “Study design”:  “In total for each participant, the script included instructions to cough 20 times, sneeze 10 times, clear throat 5 times and produce 15 sounds (explosive words, for example, “paella” and numbers as “93”).”

**2.15**

**Comment: **Methods: MINOR: Why does the Hyfe system classify every 0.5 seconds, but labeling was done every 1 second?

**Answer: **This is a misinterpretation. Hyfe uses 0.5s snippets to classify explosive sounds. Labelers were instructed to label every beginning of a cough sound, and the unit of analysis chosen was cough seconds.

**Actions: **None taken.

**2.16**

**Comment: **Methods: MAJOR: Multiple coughs may occur within a 1 second window. Why label a full 1 second window as a single true positive cough even if there could be multiple single manually labeled coughs within the 1 second? In my opinion, cough rate estimation would be more accurate if all coughs were considered rather than labeling a 1 second window of coughing as a single cough event (when estimating app performance). Is there reasoning for this in the literature? If so, please add relevant citations. If I understand correctly, it means technically you could miss all but one cough in a given 1sec window and still have very high sensitivity/specificity based on your labeling system? If this is the case, this is a major limitation. How coughs are grouped together in peals or bouts can have a significant impact on cough rate estimation. I think this discrepancy would become more evident had the system been evaluated on a cough-by-cough basis and over longer periods on patients with respiratory disease as they may tend to cough in groups in quick succession (within 1 second in many cases).

**Answer:**

 Our own data from more than 400hrs monitoring multiple patients with respiratory diseases in real-world environments shows a clear correlation between total coughs and cough seconds – this work is being prepared for publication. We are also analysing cough-seconds and the notion of bouts in the continued work. In the meantime, the objective of this work was to analyse the performance in detecting sounds, capturing and classifying coughs from solicited sounds in a controlled environment.

**Actions: **Added to the “Discussion”: “Our own data from more than 400hrs monitoring multiple patients with respiratory diseases in real-world environments shows a clear correlation between total coughs and cough seconds – this work is being prepared for publication. We are also analysing cough-seconds and the notion of bouts in the continued work. In the meantime, the objective of this work was to analyse the performance in detecting sounds, capturing and classifying coughs from solicited sounds in a controlled environment.”

**2.17**

**Comment: **Methods: MINOR: Was there any overlap in the labeling of 1 second windows i.e. 50% overlap between consecutive windows for labeling?

**Answer:** No, there was not.

**Actions: **None taken.

**2.18**

**Comment: **Methods: MAJOR: Sample Size: “385 sounds” – are you referring to cough sounds here, or a mix of both cough and non-cough? How did you determine the balance of data between cough and non-cough (speech, throat clear, noise etc.)? It seems a balance of approximately 1:2 (coughs to non-coughs) was employed. In real-life settings, over the course of a 24-hour period for example, the number of non-cough segments will far outweigh the number of cough segments if Hyfe was to continuously record audio. This does not seem to be considered here. If the data were to be more realistically balanced in this way, it may have a significant effect on the positive predictive value (PPV) of the algorithm (PPV needs to be reported). One of the biggest challenges of longer term (24 hours or more) audio-based cough monitoring is the PPV performance measure.

**Answer: **As the text states, this refers to the total number of sounds. Once more, this study did not evaluate the balance of cough and non-cough segments over a long period of time, as the objective was to only evaluate the performance of automated listening to elicited sounds, capturing and quantifying elicited coughs in controlled environments, which we achieved and described in detail.

**Actions: **None taken.

**2.19**

**Comment: **Methods: MAJOR: One of the advantages of Hyfe, reported by the authors, is that it can monitor cough rate over longer periods (>24 hours). But this study looked at 10-minute recording windows. Even taking into account that no respiratory symptoms were not noted from participants (which I think is a major limitation), I think the balance of data here are not truly representative of real-life settings especially if the authors suggest Hyfe can monitor cough rate over 24-hour periods. This is a major limitation to this evaluation study. This system needs to be evaluated over at least 24-hour periods before it can be suggested that it may perform strongly for longer term analysis in clinical settings

**Answer: **Here again, you are requesting data that exceeds the scope of this work, which was to evaluate the performance of the Hyfe system in controlled environments with solicited coughs. There is published data about the capacity of Hyfe to monitor patients for periods of months (references 12-14).

**Actions: **None taken.

**2.20**

**Comment: **Methods: MINOR: Synchronization of labels - Please provide a clearer explanation for this. Why synchronize to within 2 seconds? This seems like a long duration considering multiple explosive cough sounds may occur within 2 seconds.

**Answer: **We clarify that synchronization was done to the exact time of the sound. The text has been modified to reflect the sequential nature of changes.

**Actions: **Edits in capitalized letters: “Labels created by listeners (in Audacity) and Hyfe detected coughs were FIRST manually synchronized to within two seconds of each other. Synchronization was THEN carried out for each phone and each session separately by identifying the time offset that would align Hyfe detections with the labels and adjusting the Hyfe detection timestamps accordingly.”

**2.21**

**Comment: **Methods: MINOR: Data were discarded if they had less than 10 cough seconds of agreement. How often did that occur? How much data were discarded overall? – from Figure 1 it seems 20-25% of data were discarded due to inadequate quality. How would this reflect real-life evaluation then? The app would be unsure of about 20% of coughs at best? That seems to be a potential large margin of error. Please comment on this.

**Answer: **See the third sentence of the “Results” section for clarification: “Ten sessions did not have at least 10 sounds unanimously labeled as coughs and were also excluded, leaving 37 sessions, with 672 unanimously-labeled cough-seconds, and 1,007 non-cough seconds for the final performance evaluation.”

 Additionally, the goal of this work was not to evaluate the app in real-life and a separate work - app’s evaluation publication is in progress.

**Actions: **None taken.

**2.22**

**Comment: **Methods: MAJOR: Was the 4-tier or 6-tier labeling SOP used? This is quite confusing for the reader. If 6-tier is superior in the opinion of the authors, why not revert and apply it to all data and re-run the analysis? I understand it may mean re-training the CNN but if it will be beneficial as mentioned by the others, then it should be reported here.

**Answer: **4-tier SOP was used in this study. The findings presented here informed further changes to the SOP, resulting in the 6-tier SOP. This work was described as performance evaluation of the app and its result was also the updated SOP. We will be presenting 6-tier SOP results and further updates to the SOP in other soon upcoming publications, as it was applied in multiple studies done afterwards.

**Actions: **None taken.

**2.23**

**Comment: **Methods: MINOR: “Because the utility of a cough monitor is not in noting individual coughs but rather in tracking cough rates…”. Surely identifying singular coughs should be the goal to obtain the most accurate estimation of cough rate? If not, you could hypothetically be detecting false positives and false negatives, but still hit the correct cough rate. This could affect the analysis of how cough rate changes over time throughout a 24-hour period, for example, if it were of interest. Please provide a stronger argument for this.

**Answer:** The Hyfe Cough Monitoring System recognizes and timestamps users’ coughs as they occur. To manage the challenges of distinguishing coughs that happen in rapid succession (bouts), these timestamps are converted into cough-seconds as the basic unit of analysis; a cough-second is a second during which at least one cough occurs. The endpoint of our validation trial consists of hourly tabulations of these cough-seconds for each subject.

**Actions: **None taken.

**2.24**

**Comment: **Results: MAJOR: Please report SNR for audio sessions in comparison to cough. It may help the reader understand the poor performance of sessions 2, 17 and 38. Real-life recordings will be full of different types and levels of noise. If it is only possible to evaluate on audio within controlled environments, then the authors should augment the data by adding various different types and levels of noise to the data to replicate real-life use.

**Answer: **The goal of this study was not to evaluate Hyfe in real life environments. Ongoing studies validate the technology in real-life environments and publications will prove that in the near future.

**Actions: **None taken.

**2.25**

**Comment: **Results: MINOR: What were the uncommon acoustic characteristics associated with Sessions 2 and 17? An additional figure with time domain plot as well as a spectrogram plot of coughs from these participants would be useful here for the reader to understand why the system did not perform as well for these participants.

**Answer: **It is mentioned in the manuscript that sessions 2 and 17 had biphasic decibel peaks and different spectrogram features. This was a result of, for example, multiple solicited coughs being produced and the nature of the solicited sound, even though the script instructed a single cough. 

**Actions: **None taken.

**2.26**

**Comment: **Results: MINOR: Figure 3 image quality should be improved.

**Answer: **“Figure 3” quality is improved.

**Actions: **“Figure 3” is replaced with a better quality image.

**2.27**

**Comment: **Results: MINOR: “Sensitivity for the Session 20 was not evaluated because this was a patient with refractory chronic cough that generated hundreds of out-of-script, making timestamping impossible.” – Please elaborate further. Were there too many coughs? One would assume the data from this participant would have been very interesting to analyze and run through the Hyfe system?

**Answer: **The cough pattern of this participant has been analyzed and is described elsewhere (Reference 13). 

 She made an attempt to collaborate in this particular experiment as well but her chronic cough resulted in hundreds of observations outside the script.

**Actions: **None taken.

**2.28**

**Comment: **Results: MINOR: Limitations: I agree with the limitations mentioned. So why evaluate the Hyfe system in a way it will rarely if not never be used in? Please comment on this.

**Answer: **Because this was not an evaluation of the system in real world environments, but of the performance of the algorithm in a controlled environment. 

**Actions: **None taken.

**2.29**

**Comment: **Results: MAJOR: Please give details of the effect the Hyfe app has on battery life. One would think a continuous audio recording will significantly drain a smartphone battery. It seems to be mentioned in a previous study (ref 13). Were there experiments done on this? If not, this analysis needs to be performed and reported in this evaluation study.

**Answer: **These details are outside the scope of this manuscript. Hyfe was successfully used by hundreds of participants in a cohort study described elsewhere with no major complaints about the battery life REF 14 in the manuscript (DOI: 10.1183/23120541.00053-2022).

**Actions: **None taken.

**2.30**

**Comment: **Results: MAJOR: A Bland Altman plot analyzing cough rate between Hyfe and manual labels is required here. Correlation analysis is not enough to convince the reader that the Hyfe system can accurately estimate cough rate (unless you were to use the linear regression equation to map Hyfe estimation to manual estimation which I don’t think is the goal here). While strong correlation is interesting to observe, simply showing the equal line on Figure 4 does not highlight if there is any bias in cough rate estimation. Please add the linear regression line and equation in Figure 4. Please also add an additional panel for a Bland Altman plot comparing the mean cough rate between manual and Hyfe to the difference in cough rate between manual and Hyfe.

**Answer: **Bland-Altman plot analyzing cough rate between Hyfe and human annotators is presented as Figure 6, also Figure 5 shows the linear analysis.

**Actions: **Added “Figure 5” and “Figure 6” to the “Results” section for linear analysis and Bland-Altman plots.

**2.31**

**Comment: **Discussion: MAJOR: Where does the score threshold of 0.85 come from? Can you report the distribution of CNN output probability scores for all label categories as supplementary material? A histogram, for example, of probability scores for each label category would be interesting to see and could enhance the reader’s understanding of the challenges in labeling category 1 and 2 sounds.

**Answer: **The threshold of 0.85 was chosen as the cut-off point as it correlates well with the cough second measure while minimizing false-positives, results from the early ROC curves have been published (REF 13 in the manuscript).

**Actions:** A separate manuscript presenting a broad range of thresholds and their impact on correlation with cough seconds and true cough counts is in preparation.

**2.32**

**Comment: **Discussion: MINOR: The discussion on inter-participant variability and the debate on using solicitated voluntary coughs as a diagnostic tool is quite interesting. Does this suggest these are most likely voluntary throat clears rather than involuntary coughs? As a result, why then prompt the user to record a voluntary cough when first using the app?

**Answer: **The discussion does not discuss throat clears and our publication does not suggest that solicited coughs are not suitable for diagnostic purposes - more research is needed in this field, and that was not the scope of this study.

**Actions: **None taken.

**2.33**

**Comment: **Discussion: MINOR: What difference will the 6-tier SOP make for training the CNN model? I assume fixed 0.5sec segments are employed for training the model so how will the duration markers be more beneficial than just the onset markers used in the 4-tier SOP labeling system?

**Answer: T**he labeling by segment, rather than by peak provides the CNN with a breadth of data around the expulsive phase. 

**Actions: **None taken.

**2.34**

**Comment: **Discussion: MAJOR: The Pearson correlation is interesting, but I disagree that it should be the primary metric of performance. I think the authors should perform a linear regression analysis also to analyze the slope of the regression line. Only using Pearson correlation does not disclose information regarding how many coughs are missed in detection. For example, you could theoretically have a Pearson coefficient of 1 but you are missing 1 cough in every 2 or 3 coughs consistently in your detection model (this would be reflected in the slope of the regression analysis). The Pearson correlation metric is one of a group of metrics that should be considered in my opinion. I would like to see linear regression analysis, absolute percentage error according to the total number of coughs per participant and a Bland Altman analysis.

**Answer: **We are adding “Table 3” to cover linear model parameter estimates: Pearson correlation, intercept and slope for both phones used.

**Actions: "**Table 3” added to show the linear analysis on model parameter estimates for both phones used.

**2.35**

**Comment: **Discussion: MAJOR: “…Hyfe’s performance is adequate to be used in most clinical and research contexts.” This study does not suggest this in my opinion if the data were collected in controlled environments, was not evaluated on real respiratory patients and the smartphones were not evaluated in multiple different positions/locations.

**Answer: **We agree, this phrase was overstated. Editing the manuscript.

**Actions: **Last paragraph of the “Discussion” was edited to: “Hyfe’s performance is adequate to proceed to validation in clinical context”.

**2.36**

**Comment: **Other Comments: MAJOR: While I understand the data were obtained in controlled environments, in my opinion, more data are required to obtain a more realistic measure of performance in order to suggest Hyfe could be used in clinical settings. This includes more data using different smartphone locations (in user’s pocket, bag etc.), more realistic data with different types and levels of noise (including muffled coughs due to hand/mask covering) and I think it should be evaluated on a cough-by-cough basis rather 1 second windows as a form of cough rate.

**Answer: **This was not the scope of this study and we have changed the overstated “adequate in..clinical context” to “adequate to proceed to validation…”. All the points mentioned in this comment are being addressed in a separate clinical validation study.

**Actions: **None taken.

**2.37**

**Comment: **Other Comments: MINOR: How does the app know if it is the user who coughed and not a person beside them in a crowded area? Has this been taken into account within the CNN training data? Please comment on this.

**Answer: **This was not evaluated in this study, as the scope was just to evaluate the algorithm in detecting and recording solicited coughs in a controlled environment. 

**Actions: **None taken.

**2.38**

**Comment: **Other Comments: MINOR: Does the Hyfe app still record audio when the user is on a phone call or sending voice messages?

**Answer:** This was not evaluated and not the scope of this study and the manuscript. Just for the interest - the version of the app tested in this study would have not recorded coughs on a call/voice message.

**Actions: **None taken.

**2.39**

**Comment: **Other Comments: MINOR: Does the app work without internet connectivity?

**Answer: **The connectivity details were not in the scope of this study, therefore, no information provided in the manuscript. Just for the interest - the version of the app tested in this study works without internet connectivity to capture the sounds, but not analyze them (internet connectivity was needed). The most recent version works fully on-device, with no connectivity needed to both capture and analyze the sounds.

**Actions: **None taken.

**2.40**

**Comment: **Other Comments: MINOR: SOP4 Supplementary Document: Page 4, Section 3, bullet 3 – should that read as “…at the end of the expulsive phase..”?

**Answer: **This is correct, a spelling mistake was made in “expulsive”.

**Actions: **Correcting the spelling mistake.

**2.41**

**Comment: **Other Comments: MINOR: SOP4 Supplementary Document: I think reporting a cough as having one sound or two sounds is a little confusing. Consider moving Fig. 5 and the paragraph above it to before Fig. 3. Start with showing the sound, explaining the three phases of the cough sound event and then describe in more detail the physiology behind it. Fig. 3 panel B needs to highlight that the voiced phase is missing. It is a little confusing to just say A has a “double” sound and B has a “single sound”.

**Answer: **We appreciate your feedback. With the SOP being updated, confusing parts have been addressed in newer editions.

**Actions:** None taken.

**2.42**

**Comment: **Other Comments: MAJOR: SOP4 Supplementary Document: Page 8, Table 1 – according to the 4-tier process, the label 2 may be due to the phone located in the user’s pocket which, I assume, will be a vastly common occurrence if this app were to be used in cough clinical applications. In this study label 2 coughs weren’t considered in the app evaluation. Why not? I think it is essential particularly for an app-based cough monitoring system to be evaluated on recordings when the phone is in the user’s pocket or bag for example.

**Answer:** The scope of this research was not to evaluate the app in real-world environments, just the performance of the algorithm with solicited sounds in controlled environments. Further studies and validation of the system address these concerns mentioned in your comment.

**Actions: **None taken.

**2.43**

**Comment: **Other Comments: MINOR: SOP4 Supplementary Document: Page 8, Labeling Tips – I think the label 2 may highlight a potential limitation of the Hyfe system. It is indicating to the reader that the smartphone needs to be in close proximity to the user. Coughs may sound “distant” even if the user is coughing. Please elaborate what “distant” means here. Is this related to the audio amplitude of the cough sound? Certain types of coughs may have lower amplitudes, particularly if a very ill patient has exhausted their respiratory strength.

**Answer: **“Distant” in the 4-tier SOP meant subjectively distant sound (compared to surrounding sound levels both audibly and visually in the recording that is being analysed). This study was not meant to evaluate the system in clinical settings, we will be reporting on system performance with low amplitude coughs in the upcoming publications from our validation studies.

**Actions: **None taken.

**2.44**

**Comment: **Other Comments: MAJOR: The smartphone can be located on a table with many other people in the user’s vicinity. How do you know it is the user’s cough? This could be a major limitation. Particularly if the intention is to detect subtle changes in cough rate. There are many external factors when using an app-based audio cough detection system that can affect cough rate estimation. I don’t think the authors make it clear how they will evaluate this or can overcome such challenges using this system.

**Answer: **Differentiating a cougher from the next one was not the scope of this study - this study was to evaluate the performance of the algorithm in very controlled environments - when a script is followed and the sound-producing person is known. In our validation studies and publications concerning real-world environments, all details will be provided.

**Actions: **None taken.

**2.45**

**Comment: **Other Comments: MINOR: If possible, it would be very beneficial to have examples of the sounds that were recorded in the controlled environment for readers to listen to.

**Answer: **You are requesting the sharing of recordings from participants in a trial. Following good practices, the informed consent form states that “only the study personnel will have access to the recordings”. This will not be possible.

**Actions: **None taken.

**2.46**

**Comment: **Is the work clearly and accurately presented and does it cite the current literature? No

**Answer:** It would be important for the reviewer to back this assessment with specific examples of where the literature was not cited or done so inappropriately. 

**2.47**

**Comment: **Is the study design appropriate and is the work technically sound? No

**Answer: **The vast majority of the comments by this reviewer were for broader scope than the primary objective of the study described in the manuscript.

**2.48**

**Comment: **Are sufficient details of methods and analysis provided to allow replication by others? No

**Answer: **The Methodology section is now updated and the full dataset and code are publicly available.

**2.49**

**Comment:** If applicable, is the statistical analysis and its interpretation appropriate? Partly

**Answer:** Many of the comments by this reviewer were for broader scope than the primary objective of this study described in the manuscript.

**2.50**

**Comment: **Are all the source data underlying the results available to ensure full reproducibility? No

**Answer: **The full data has been made available with the original version. 

**2.51**

**Comment: **Are the conclusions drawn adequately supported by the results? Partly

**Answer:** Many of the comments by this reviewer were for broader scope than the primary objective of this study described in the manuscript.

**2.52**

**Comment: **Competing Interests. No competing interests were disclosed by the reviewer.

**Answer: **This is outrageous and akin to reviewer misconduct as stated above. Please provide a full disclosure of your economic CoI or recuse.

# Reviewer report: Reviewer response for version 1

**DOI:** 10.5256/f1000research.134609.r146174

**Published:** 2022-08-16

**Authors:** Vasileios Papapanagiotou

**Affiliations:** 1 Electrical and Computer Engineering Department, Aristotle University of Thessaloniki, Thessaloniki, Greece

The paper presents an evaluation study for Hyfe. Hyfe is a smartphone app that captures audio and automatically detects coughs using machine learning methods. In this work, authors captured audio using 3 sources (an mp3 recorder for ground truth and two phones running Hyfe). Then, 3 annotators created ground truth annotations, against which Hyfe detections were compared.

 The dataset includes 10-minute recordings were multiple sounds were performed in randomized sequences. The recordings were performed in complete silence. Evaluation results show high effectiveness, in particular 91% sensitivity and 98% specificity on 1,679 1-second windows.

**Questions:**

- In section “Methods”, subsection “Automated cough detection system”: it is mentioned that 0.5 second sound snippets are extracted and analyzed. Why was this not followed for the study and 1-second windows were used instead?
- In section “Methods”, subsection “study design” (last paragraph): how exactly were the coughs annotated? Is it with start & stop timestamp per cough? If so, please state clearly. Figure 5 is helpful and perhaps should be presented much earlier in the manuscript. Also, did each of the 3 medically trained researchers perform this process? Also, what is a “sound” in this paragraph?
- Inter-annotator agreement would be very interesting to know.
- In section “Methods”, subsection “Data processing and analysis”: what does the “within two seconds of each other” mean? Was the maximum allowed offset between the two recordings limited by 2 seconds? If so, why? Please clarify.
- In section “Methods”, subsection “Data processing and analysis”: authors mention “if multiple explosive sounds occurred within a cough-second”. Given that Hyfe uses 0.5 second snippets and authors used 1-second windows, exactly 2 Hyfe predictions should always “fall” within one 1-second window. If so, why do the authors phrase this this way?
- Section “Methods”, subsection “Data processing and analysis”: was the 6-tier SOP used after all? If not, why is it mentioned? (mentioning it while it's not being used in the work can be confusing to the reader).
- Section “Methods”, subsection “Data processing and analysis” (last paragraph): authors mention that cough rates are of high interest in such applications. However, the data collection protocol forces some strict limitations, e.g., a 5-second silence between each activity.
- In section “Methods”, subsection “Sample size”: authors mention that “at least 385 sounds…” What is a sound in this context? Is it a 1-second window?
- In section “Results”, authors mention that in total 37 sessions were used, each being a 10-minute recording. This corresponds to 370 minutes, i.e., 22,200 seconds. However, authors also mention that the 37 sessions resulted in 672 + 1,007 = 1,679 seconds were used in the evaluation. It is not clear how the discrepancy from 22,200 to 1,679 seconds happens.
- Figure 2: what is the unit and scale for the x-axis (for both subfigures)?
- Figure 3: image quality should be improved.
- Figure 4: Authors mention that 5 seconds of silence were required (at least) between each activity. Given at least 1 second for cough, this yields a pattern of 6 seconds, and this yields a maximum of 10 coughs per minute. If so, how are values larger than 10 obtained in this plot?
- Discussion: continuous 24-hour audio recording can have a significant effect on battery consumption, this is an important limitation of the method. Also, the audio caused by the user when holding and using the phone (while it is recording audio) is also critical in evaluation of the Hyfe effectiveness.
- Discussion: "we believe that labeling cough duration rather than just its beginning has more value in further training Hyfe’s AI model". Since there is no description of the Hyfe's algorithm in the paper or any relevant experiment, this argument could be debated. 

Is the work clearly and accurately presented and does it cite the current literature?

Yes

If applicable, is the statistical analysis and its interpretation appropriate?

Not applicable

Are all the source data underlying the results available to ensure full reproducibility?

No

Is the study design appropriate and is the work technically sound?

Yes

Are the conclusions drawn adequately supported by the results?

Partly

Are sufficient details of methods and analysis provided to allow replication by others?

Yes

Reviewer Expertise:

digital signal processing, machine learning, wearables, eating behaviour

I confirm that I have read this submission and believe that I have an appropriate level of expertise to confirm that it is of an acceptable scientific standard, however I have significant reservations, as outlined above.

# Response

**Published:** 2023-04-27

**Authors:** Mindaugas Galvosas

**1.1**

**Comment:** In section “Methods”, subsection “Automated cough detection system”: it is mentioned that 0.5 second sound snippets are extracted and analyzed. Why was this not followed for the study and 1-second windows were used instead?

**Answer: **Thank you for taking the time to review this paper and provide comments. 

 Our algorithm at the time of this study was trained on a database of more than 200M of 0.5s sound snippets that are cough and cough-like sounds, collected in real life environments. Therefore, this performance evaluation also evaluated 0.5s sound snippets. Continuing our work, we are exploring the capture and analysis of 1s sound snippets, however, it was not the scope of the work described in this manuscript.

**Actions: **None taken.

**1.2**

**Comment: **In section “Methods”, subsection “study design” (last paragraph): how exactly were the coughs annotated? Is it with start & stop timestamp per cough? If so, please state clearly. Figure 5 is helpful and perhaps should be presented much earlier in the manuscript. Also, did each of the 3 medically trained researchers perform this process? Also, what is a “sound” in this paragraph?

**Answer: **In the performance evaluation study described in this manuscript, coughs were annotated following the 4-tier SOP – which instructed to indicate just the beginning of the cough sound. Further work led to developing a 6-tier and most recently – a multi-tier SOP which instructs cough and other respiratory sound annotation from the beginning to the end, taking both audio and visual (spectrogram) inputs to determine those marks.

 For this study – each of the 3 medically trained researchers annotated every cough and cough-like sound following the 4-tier SOP.

**Actions: **Added a linear analysis (Figure 6) plot and a percentage error Bland-Altman plot (Figure 7) to the section “Results”.

**1.3**

**Comment: **Inter-annotator agreement would be very interesting to know.

**Answer: **Thank you. We agree this is an interesting point to look at. There is a separate full paper in preparation looking specifically at this with a whole different dataset. 

**Actions: **None taken.

**1.4**

**Comment: **In section “Methods”, subsection “Data processing and analysis”: what does the “within two seconds of each other” mean? Was the maximum allowed offset between the two recordings limited by 2 seconds? If so, why? Please clarify.

**Answer: **The manual offset was made to align the data annotation by different labelers (in an mp3 audio recording) and Hyfe timestamps on the smartphones. Due to the automated script setting, which had five second gaps between every solicited sound, two second differences were not outside the five second gap.

**Actions:** Added “(as this was within the silent time of five seconds between the solicited sounds in the automated script)” in the “Data processing and analysis” subsection to clarify the manual offset of the two-second time frame.

**1.5**

**Comment: **In section “Methods”, subsection “Data processing and analysis”: authors mention “if multiple explosive sounds occurred within a cough-second”. Given that Hyfe uses 0.5 second snippets and authors used 1-second windows, exactly 2 Hyfe predictions should always “fall” within one 1-second window. If so, why do the authors phrase this this way

**Answer: **2 Hyfe predictions will always be found within 1 second window but 'multiple explosive sounds' refers to a prediction of a 0.5sec snippet that contains at least 1 cough, which doesn't happen every time. Therefore, the phrasing is appropriate.

**Actions: **None taken.

**1.6**

**Comment: **Section “Methods”, subsection “Data processing and analysis”: was the 6-tier SOP used after all? If not, why is it mentioned? (mentioning it while it's not being used in the work can be confusing to the reader).

**Answer: **The text states “This analysis further informed the SOP… leading to the most recent version – the 6-tier SOP…” and we believe there should be no confusion here.

**Actions: **None taken.

**1.7**

**Comment: **Section “Methods”, subsection “Data processing and analysis” (last paragraph): authors mention that cough rates are of high interest in such applications. However, the data collection protocol forces some strict limitations, e.g., a 5-second silence between each activity.

**Answer: **While rates indeed remain interesting, calculating them was outside the scope of this performance evaluation, nor is it possible with the methodology that was followed. The main purpose of this work was to evaluate the performance of the system capturing and recording solicited coughs and cough-like sounds in controlled environments.

**Actions: **None taken.

**1.8**

**Comment: **In section “Methods”, subsection “Sample size”: authors mention that “at least 385 sounds…” What is a sound in this context? Is it a 1-second window?

**Answer: **Sound refers to any cough or other sound produced by the study participants according to the script provided.

**Actions: **None taken.

**1.9**

**Comment: **In section “Results”, authors mention that in total 37 sessions were used, each being a 10-minute recording. This corresponds to 370 minutes, i.e., 22,200 seconds. However, authors also mention that the 37 sessions resulted in 672 + 1,007 = 1,679 seconds were used in the evaluation. It is not clear how the discrepancy from 22,200 to 1,679 seconds happens.

**Answer: **1,679 was the number of solicited sounds that were evaluated, not the total number of seconds of recording for all participants. Further explanation is provided in the “Study design” section.

**Actions:  **Improved “Study design” section: “Participants were asked to produce a series of solicited sounds by reading a provided script, while being recorded with an MP3 recorder and monitored by Hyfe on two identical smartphones. The phones and recorder were placed on a table at approximately 50 cm from the participants, with microphones oriented towards them. A pre-generated computer script instructed participants to produce a series of 46 sounds, of which 18 were coughs, the rest consisted of solicited sneezes, throat clearings, spoken letters or words in the same 10 minutes. Participants were instructed to cough once every time they were prompted by the script to do so. In total for each participant, the script included instructions to cough 20 (18 as individual coughs and 2 in the literary text) times, sneeze 10 times, clear throat 5 times and produce 15 sounds (explosive words, for example, “paella” and numbers as “93”). “

**1.10**

**Comment: **Figure 2: what is the unit and scale for the x-axis (for both subfigures)?

**Answer: **The name of the x-axis in this version is called “frequency of observation”, however, after another review with our data science team, we have agreed to change this Figure 2 to a new visual - newly submitted histograms in Figure 2, which convey the same information on specificity and sensitivity for both phones more clearly and, in our opinion, more efficiently.

**Actions: **“Figure 2” updated to a new visual. The “Figure 2” is also now accompanied by the “Table 2: Summary statistics on sensitivity and specificity for both phones used”.

**1.11**

**Comment:** Figure 3: image quality should be improved.

**Answer: **Thank you for noting, the image will be replaced with a better quality file.

**Actions: **New image generated and uploaded (Figure 3).

**1.12**

**Comment: **Figure 4: Authors mention that 5 seconds of silence were required (at least) between each activity. Given at least 1 second for cough, this yields a pattern of 6 seconds, and this yields a maximum of 10 coughs per minute. If so, how are values larger than 10 obtained in this plot?

**Answer: **Some participants were instructed to cough once, however, they produced two coughs. These coughs were annotated by the labelers and also picked up by Hyfe, when they happened with at least  0.5s of separation.

**Actions: **None taken.

**1.13**

**Comment: **Discussion: continuous 24-hour audio recording can have a significant effect on battery consumption, this is an important limitation of the method. Also, the audio caused by the user when holding and using the phone (while it is recording audio) is also critical in evaluation of the Hyfe effectiveness.

**Answer: **This is a valid point; however, this performance evaluation was done in laboratory settings and was not meant to evaluate battery usage and performance. Additionally, directionality of the audio source was not evaluated by this study and is being evaluated with the continued studies.

**Actions: **None taken.

**1.14**

**Comment: **Discussion: "we believe that labeling cough duration rather than just its beginning has more value in further training Hyfe’s AI model". Since there is no description of the Hyfe's algorithm in the paper or any relevant experiment, this argument could be debated.

**Answer: **For model training purposes we have seen that labeling the full duration of the sound is a better way of annotating data, ensuring that the algorithm is being trained on the full duration of cough sound. As convolutional neural networks are employed by Hyfe - they learn by example. As long as the training data is relatively unbiased and representative, a neural net can identify a “feature” (such as the acoustic signature of a cough) in a myriad of samples, even if those samples do not resemble each other.

**Actions: **Added to the “Discussion”: “As convolutional neural networks are employed by Hyfe - they learn by example. As long as the training data is relatively unbiased and representative, a neural net can identify a “feature” (such as the acoustic signature of a cough) in a myriad of samples, even if those samples do not resemble each other.”

**1.15**

**Comment: **Are all the source data underlying the results available to ensure full reproducibility? – replied “No”.

**Answer: **All source data is updated and made available to reproduce all the analysis presented in the manuscript.

**Actions: **None taken.

**1.16**

**Comment: **Are the conclusions drawn adequately supported by the results? – replied “Partly”

**Answer: **We believe that the proposed changes will strengthen the conclusions drawn from the results, thank you for your input and feedback.

**Actions: **None taken.
